# A high-performance fluorescent hybrid material for fluorometric detection and removal of toxic Pb(ii) ions from aqueous media: performance and challenges†

**DOI:** 10.1039/d2ra07651a

**Published:** 2023-01-20

**Authors:** Abdulrazzak Abdullah, Ahmed Nuri Kursunlu, Ersin Guler

**Affiliations:** a Department of Chemistry, Selcuk University Campus 42031 Konya Turkey ankursunlu@hotmail.com +90 332 223 39 02

## Abstract

Lead(ii) is an extremely toxic heavy metal ion that causes various health problems that are difficult to recover from in many developing countries of the world. Fluorescence-based nanosensors have amazing characteristics such as high sensitivity/selectivity, portability, low detection limit, rapid on-site usability, low cost and capability for removal of heavy metal ions. In this paper, a new fluorescent hybrid material based on silica gel (Bodipy-Si) was developed *via* a click reaction between alkyne-terminal silica gel and azido-terminal Bodipy. The solid support surface was characterized by various techniques such as SEM, FT-IR, *etc.* The adsorption and fluorometric properties of the fluorescent nanoparticles were also examined using atomic absorption and fluorescence spectroscopies, and in the presence of metal ions, respectively. The results indicated that the prepared hybrid-fluorescent nanoparticles can be used in the removal and detection of toxic Pb(ii) ions. The limit of detection (LOD) was determined from the fluorescence data as 1.55 × 10^−7^ M and the maximum adsorption capacity was examined by AAS. The complexometric interactions between Pb(ii) and Bodipy-Si affect the adsorptions of the Pb(ii) metal ion at various concentrations.

## Abstract 2

The hybrid surface, Bodipy-Si, was prepared for detection and adsorption of Pb(ii) ions.

## Graphical abstract

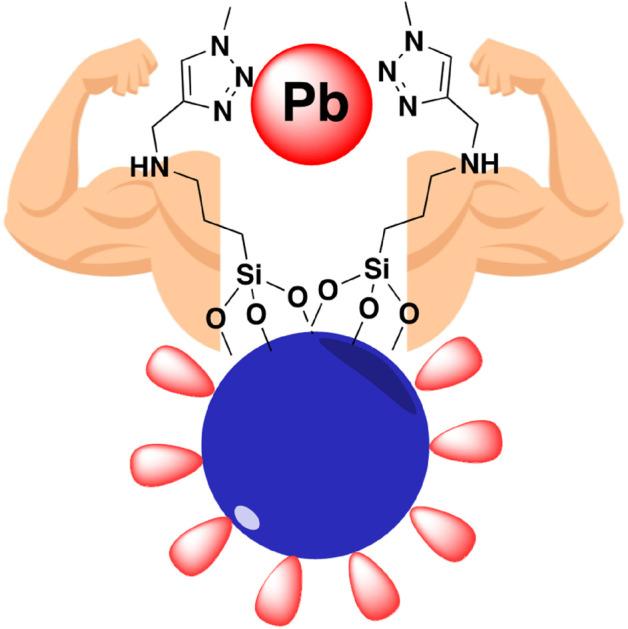

## 1. Introduction

The rapid increase in industrialization and the human population is causing serious environmental problems. It is known that the presence of chemicals such as toxic heavy metal ions, micro-pollutants, dyestuffs, phenols, pesticides, detergents, and other persistent organic pollutants causes widespread water pollution in different parts of the world.^1–7^ The discharge of these toxic pollutants into natural waters greatly affects the ecological balance and causes harmful effects on vegetation and animals. Therefore, it is important to treat contaminated water and wastewater before discharge into the environment.^8–10^ One of the most important causes of water and environmental pollution is the mixing of toxic metals into the water. Heavy metal ions are not biodegradable and easily accumulate in living organisms in the food chain, leading to various diseases or disorders such as tremors, kidney lesions and even cancer.^11,12^ Although some heavy metals are necessary for our bodies, they become toxic above the safe limit. Some heavy metals are known to cause great harm to the human body regardless of their concentration.^13–16^

Among these ions, lead is a highly toxic and non-degradable heavy metal that is the most toxic metal after arsenic due to its hazardous effects on living organisms.^17–19^ It has no beneficial role in biological systems and is harmful to plants, animals, and humans. People can be exposed to lead by breathing in lead-contaminated dust particles or from food, water, and dyestuffs.^20,21^

Lead poisoning in humans directly affects the nervous system and all organs that the US Environmental Protection Agency and the World Health Organization have specified the maximum allowable concentrations of lead in drinking water as 0.015 mg L^−1^ and 0.01 mg L^−1^, respectively.^22,23^ Wastewater treatment has become an industry that contributes to the prevention of environmental pollution.^24–26^ It is important to use cost-effective and environmentally friendly methods in wastewater treatment. Today, various methods are used for the detection and removal of a heavy metal ion such as chemical precipitation, ion exchange, adsorption, reverse osmosis, and membrane filtration.^27–30^ Among the mentioned methods, adsorption is the most preferred method due to its ease of use, low cost, and high efficiency.^31^ High adsorption capacity and reusability should be considered while selecting the adsorbent in the adsorption method just as the functionalized silica gel nanoparticles.^32–34^ Recently, it has been desired that the silica gel materials used in the removal of heavy metals have a fluorescence property.^35–40^

All this information was evaluated, and this study aimed to prepare a new fluorescent hybrid material. A Bodipy derivative and the modified silica gel were preferred as the source of fluorescent character and adsorbent surface, respectively. The effective using of the prepared material was investigated in both the recognition and removal of metal ions. According to the results obtained, it was determined that the newly prepared fluorescent nanoparticle could selectively and sensitively detect lead ions and could be used effectively in the removal of these ions. Therefore, this paper will be a challenge against rivals and useful for science world researchers working on the improvement of fluorescent hybrid material to sensitively detect and removal of lead ions in various mediums.

## 2. Experimental section

### 2.1. Chemicals and apparatus

The NMR spectra (^1^H and ^13^C) of the synthesized organic compounds were performed using a nuclear magnetic resonance spectrometer (Varian 400 MHz). The emission spectra and the linked spectroscopic measurements were performed by a PerkinElmer LS 55 spectrofluorometer. The SEM images, FT-IR spectra and elemental analysis were performed with a HITACHI (SU5000), a Bruker Fourier Transform Infrared (ATR) and a Leco CHNS 932, respectively. The remaining lead ions in the suspension were determined by Analytic Jena, Contr AA 300 spectrophotometer using the atomic absorption values.

For the ordering of the used chemicals, various companies were preferred. The silica gel (70–230 mesh) used as both purification (column chromatography) and adsorbent materials was purchased from Fluka (Switzerland). Sodium azide, d-chloroform (CDCl_3_), 3-aminopropyltrimethoxysilane (APTMS, 97%), propargylamine, triethylamine, 2,4-dimethyl-3-ethylpyrrole, borontrifluoride diethyl etherate, *N*,*N*-diisopropylethylamine (DIPEA), 4-(chloromethyl)benzyl chloride, sodium ascorbate, copper(ii) sulphate were provided from Sigma-Aldrich. Solvents (dichloromethane, ethyl alcohol, toluene, petroleum ether (40–60%), *N*,*N*-dimethylformamide) and the metal nitrate salts were purchased from Merck Company (Germany, Darmstadt).

### 2.2. Modifications of the activated silica gel

**Scheme 1:**
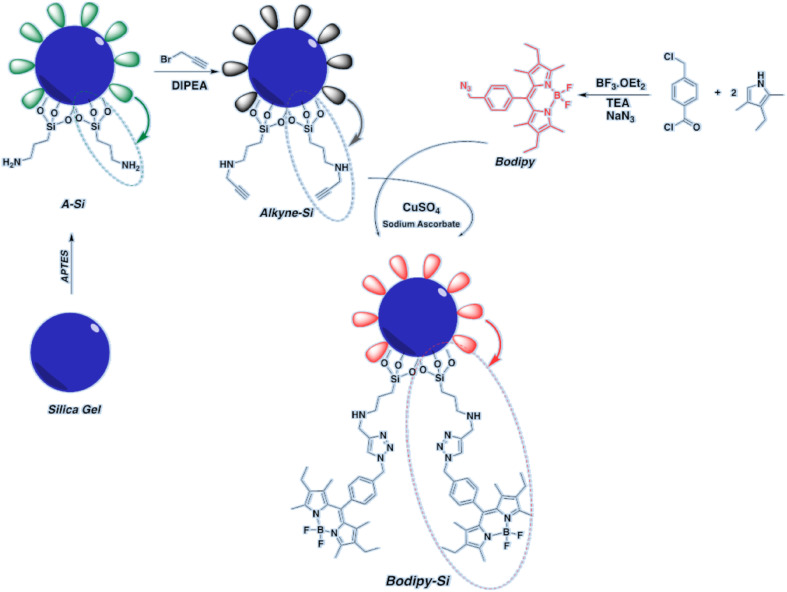
The preparing route of Bodipy-Si nanoparticle.

The activated raw silica gel, the silica gel modified with APTMS (A-Si) and Bodipy derivatives were prepared according to known synthesis procedures.^41^ The alkyne terminal silica gel (Alkyne-Si) was obtained using DIPEA from a reaction between propargylamine and A-Si.^42^ The target fluorescent nanoparticle (Bodipy-Si) was prepared from Alkyne-Si and Bodipy by the classic click reaction principle in the presence of sodium ascorbate and CuSO_4_ as a catalyst (Scheme 1). Bodipy-Si was washed respectively with ethyl alcohol, pure water and acetone and dried in a vacuum at 50 °C.

### 2.3. Selectivity and sensitivity studies of metal ion

The solutions of the metal ions were prepared in pure water (1 × 10^−5^ M) from the nitrate salts for fluorescence measurements. The stock suspension solution of Bodipy-Si was also prepared using 0.1 g of the targeted nanoparticle dispersed into 1 L of acetonitrile. A mixture of Bodipy-Si suspension (3 mL) and metal solutions (0.4 mL) was shaken into a centrifuge tube for 45 min at room temperature and more then, the fluorescent measurements were carried out. The competing ion studies of Bodipy-Si were carried out in the presence of test metal ions by various concentrations of the competing ion and the optimum pH values of the suspension solutions were adjusted with NaOH or HNO_3_ solutions.

## 3. Results and discussions

### 3.1. Characterizations

**Fig. 1:**
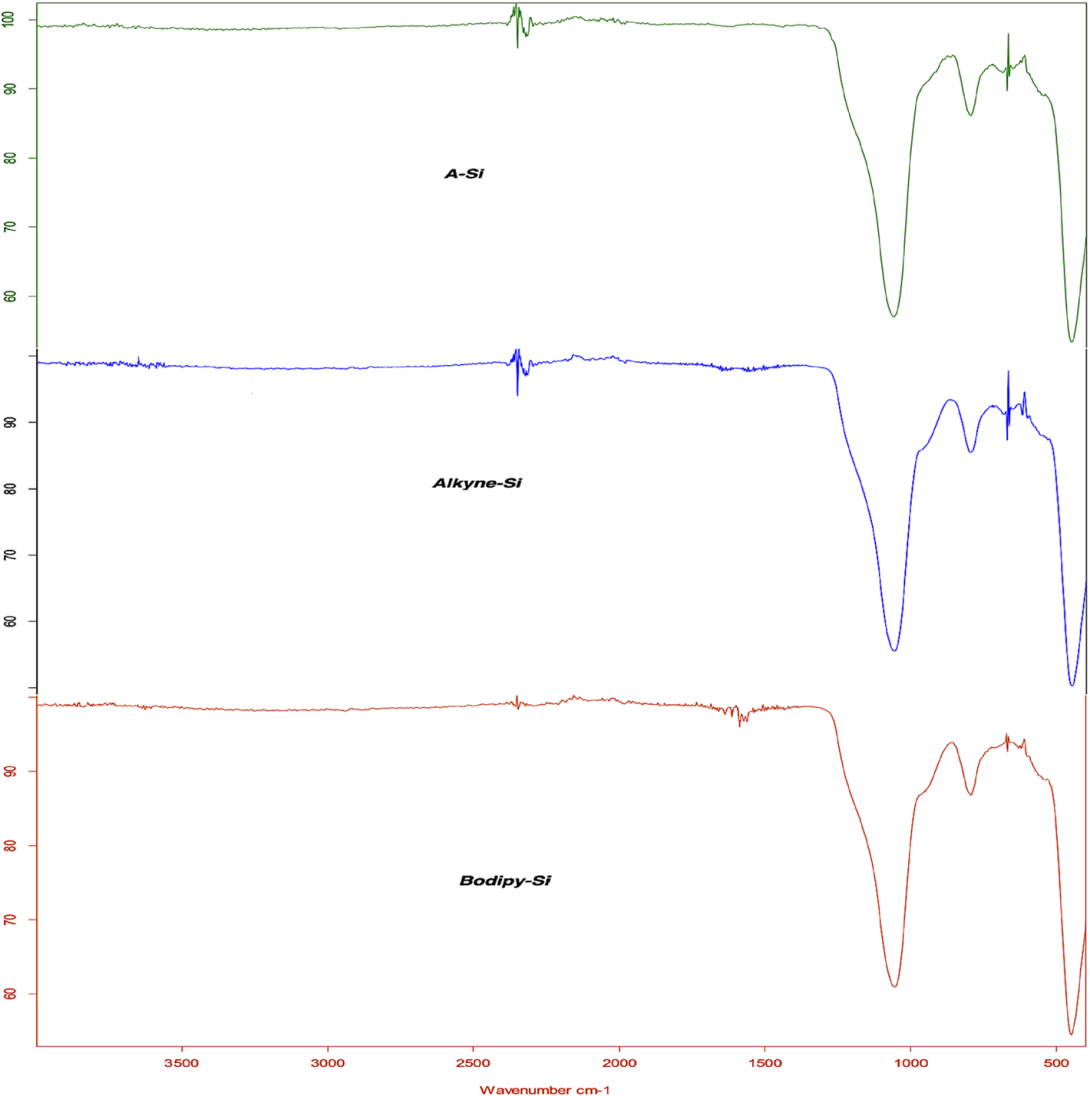
FT-IR spectra of A-Si, Alkyne-Si, Bodipy-Si and main differences among solid support surfaces.

The FTIR spectra of the functionalized support surfaces (A-Si, Alkyne-Si and Bodipy-Si) can be seen as the overlapped form in Fig. 1. The specific peaks observed around 797 and 1057 cm^−1^ in all curves assign to the asymmetric and symmetric stretching vibrations of Si–O bonding, respectively.^43^ The strong-dominant peaks overshadowed the new peaks assigned several functional groups after the transformation of Alkyne-Si and Bodipy-Si. Actually, the peak overshadowing process is well known for solid support surfaces and these peak intensities vary according to the binding percentage of the organic groups attached to the surface.^44^ However, small changes in the spectra and shifts in the main vibrations show a surface modification. So, multi vibration peaks between 1500 and 1600 cm^−1^ in the spectrum of Bodipy-Si can be attributed to the C=C and C=N stretching vibrations in modification agents (Bodipy and others). Moreover, the new vibrations around 2900 cm^−1^ in the spectra of Alkyne-Si and Bodipy-Si also assign to C–H stretching of aromatic or aliphatic fragments. On the other hand, the dominant broad peaks of the silica gel surface slightly shifted to lower frequencies. All small, however, the visible changes support to successful anchoring of both amino-alkyne and Bodipy moieties on the A-Si surface.

**Fig. 2:**
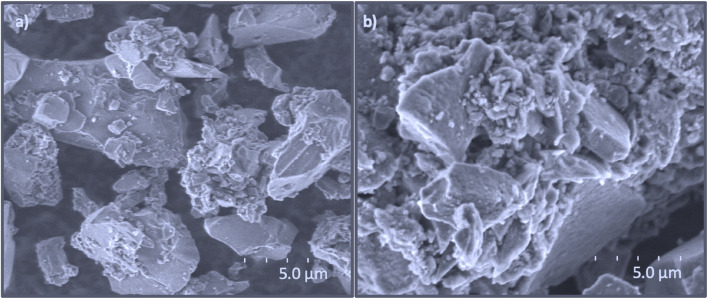
The surfaces images of (a) Alkyne-Si and (b) Bodipy-Si on SEM.

To support the successful surface modification of target adsorbents, the SEM images of Alkyne-Si and Bodipy-Si were performed. As indicated in Fig. 2a, the Alkyne-Si material has a smoother surface than the surface of Bodipy-Si (Fig. 2b) and the morphology of Bodipy-Si shows a more aggregation form. The SEM images proved that the chemical immobilization of Bodipy derivative was carried out successfully onto the functional silica gel solid support prepared by the alkyne-amino and APMTS organic groups.

### 3.2. Sensitivity/selectivity toward metal cations using fluorimetry

**Fig. 3:**
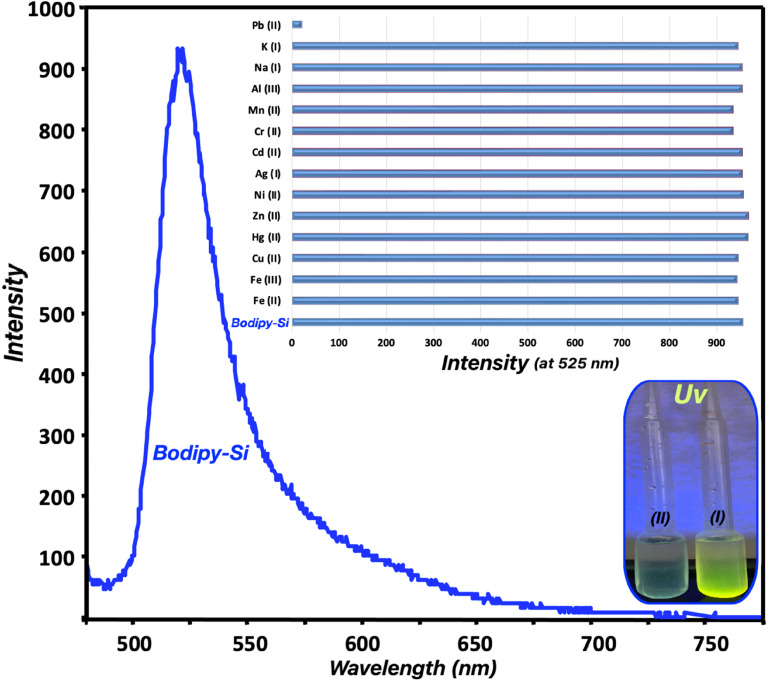
Emission spectrum of Bodipy-Si (0.1 mg mL^−1^ in methanol/water, 10/90) and emission maxima (at 525 nm) after the addition of different metal ions (Ag(i), K(i), Mn(ii), Cr(ii), Pb(ii), Cd(ii), Zn(ii), Ni(ii), Al(iii), Fe(iii), Fe(ii), Hg(ii), Cu(ii), and Na(i)) (*λ*_exc_: 450 nm) and the images of Bodipy-Si + other metal ions (I) and Bodipy-Si + Pb(ii) (II) suspensions under longwave light (365 nm).

The sensitivity studies of Bodipy-Si (0.1 mg mL^−1^) toward test metal cations were examined by the changes in emission maximum at room temperature (v/v : 10/90 in methanol : water). 3 mL of Bodipy-Si suspensions (v/v : 10/90 in methanol : water) were respectively added to metal ion solutions (Ag(i), K(i), Mn(ii), Cr(ii), Pb(ii), Cd(ii), Zn(ii), Ni(ii), Al(iii), Fe(iii), Fe(ii), Hg(ii), Cu(ii), and Na(i)) (3 mL). The emission curves of the mixtures including adsorbent and metal ions were performed in a fluorescence spectrophotometer (Fig. 3). The emission intensity of Bodipy-Si changed only in the presence of lead(ii) ions without any shift in wavelength. On the other hand, here, no an important change in the fluorescence intensity of Bodipy-Si after the addition of other metal ions. The quenching effect that occurred by the addition of lead(ii) ions can be explained by a complex interaction between the metal ion and electron-donor adsorbent. The multi-triazole units on Bodipy-Si catch lead(ii) ions and caused a fluorescence quenching effect that was assigned to a PET (photoinduced electron transfer). The sensitivity to lead(ii) ions detected by fluorescence spectroscopy was also supported by the naked-eye and Uv-vis lamp. These visual results show that Bodipy-Si can successfully be used in the colorimetric and fluorometric detection of Pb(ii) ions in an aqueous medium.

**Fig. 4:**
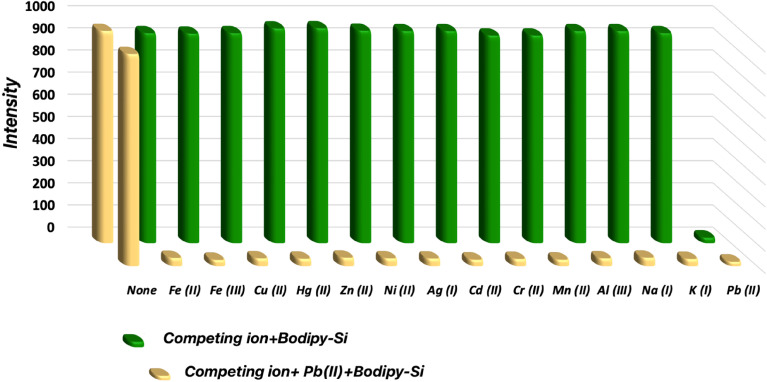
Competing ion tests of Bodipy-Si (0.1 mg mL^−1^ in methanol/water, 10/90)-Pb(ii) (5.0 × 10^−5^ M in methanol/water, 10/90) mixture (*λ*_exc_: 450 nm) in the presence of other cations (5.0 × 10^−5^ M in methanol/water, 10/90) (Ag(i), K(i), Mn(ii), Cr(ii), Cd(ii), Zn(ii), Ni(ii), Al(iii), Fe(iii), Fe(ii), Hg(ii), Cu(ii), and Na(i)).

To determine the effect of competing ions, a series of next experiments were carried out with a mixture concluding equivalent to other test metal ions + Pb(ii) + Bodipy-Si (0.1 mg mL^−1^ in methanol/water, 10/90). The fluorescence maxima at 525 nm were submitted in Fig. 4, both Pb(ii) + Bodipy-Si suspension and the competing ions + Pb(ii)+ Bodipy-Si mixture that the competing ions did not almost change the fluorescence intensity of the sensitive adsorbent/Pb(ii) system. These results support that Bodipy-Si can be selectively used in the fluorescence detection of the Pb(ii) ions. The Pb(ii) ions interact easily with the nitrogen atoms of triazole units in a complex reaction principle. In there, it can be asserted that a photoinduced electron transfer (PET) carried out with a quenching procedure between the hybrid material as a fluorophore and Pb(ii) ions upon metal–ligand interaction. Therefore, the PET generate a charge separation and redox transformation occurs between excited state and the first excited singlet state results in formation of a donor/acceptor pairs.

**Fig. 5:**
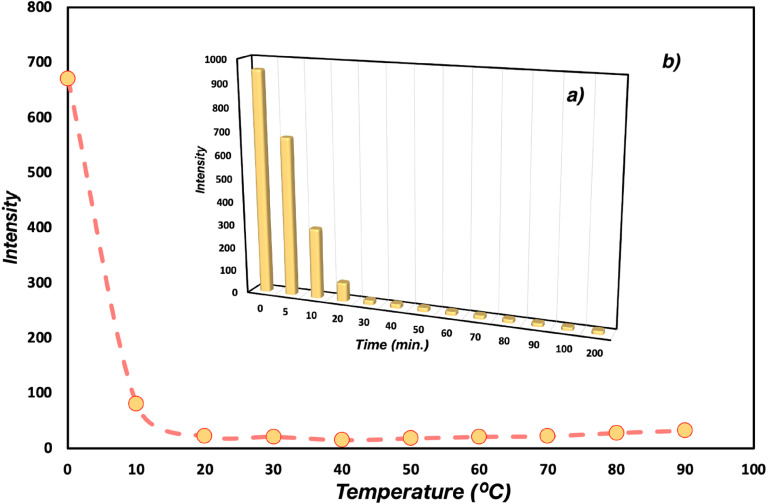
Influence of response time (a) and temperature (b) on the detection of Pb(ii) by Bodipy-Si.

The influence of response time and temperature on the detection of Pb(ii) ions was examined with the changes in the fluorescence intensity of Bodipy-Si adsorbent (Fig. 5a and b). The measurements were carried out at various minutes (0–200 min) and temperatures (0–90 °C).

The quenching effect provided by Pb(ii) ions almost stopped after the first twenty minutes and reached a constant value in the emission intensity. On the other hand, the quenching effect was still observed at temperatures above twenty degrees in the best yield, while the fluorescence intensity increases at lower temperatures. Depending on the fluorescence results, the fluorescent adsorbent can be used in the recognition of Pb(ii) ion in twenty-five minutes and temperatures above twenty degrees.

**Fig. 6:**
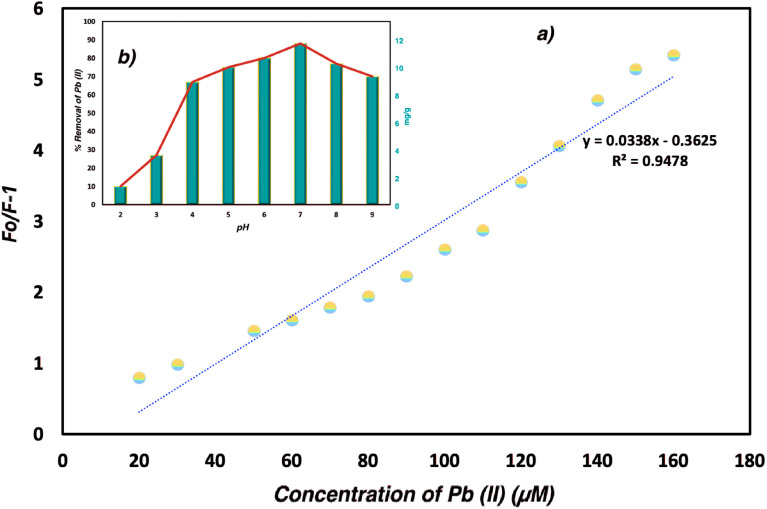
(a) The corresponding Stern–Volmer plot (*F*_0_/*F* − 1) depending on the fluorescence maxima of Bodipy-Si (*λ*_emmax_ = 525 nm) in different concentrations of Pb(ii) ions (b) Influence of adsorbent dose (mg g^−1^) and removal percent of Pb(ii) ions depending on pH values.

The Bodipy-Si fluorescence measurements were carried out with freshly prepared Pb(ii) solutions and diluted in 10 mL of distilled-water. The Stern–Volmer equation was performed with several concentrations that the quenching effect was illuminated with the binding constants. The emission maxima of Bodipy-Si suspension were obtained within the first twenty minutes after the mixing of adsorbent and Pb(ii) ions in various concentration values. Upon adding Pb(ii) ion, a substantial quenching in the emission intensity of Bodipy-Si suspension was observed and thus, the (*F*_0_/*F*) − 1 ratio increased. The LOD of Bodipy-Si was determinate as 1.55 × 10^−7^ M based on the LOD = 3SD/S. The influence of adsorbent dose (mg g^−1^) and removal percent of Pb(ii) ions was examined depending on pH values (Fig. 6a and b). In highly acidic mediums, the removal percent of Pb(ii) ions decreased while the adsorption of Bodipy-Si reaches higher values between pH: 4 and 9. As these results, Bodipy-Si can be used in a wide pH range in the detection and removal of Pb(ii) ions.

The comparison of nano probes used for the detection of Pb(ii) with our fluorescent hybrid material was shared in Table 1. As can be seen in Table 1, all other studies were performed with a fluorescence quenching just as in this paper that Bodipy-Si has a higher performance than many references with low detection limit. Moreover, this detection and removal procedure is a cheaper, easier method to prepare and portable.

**Table 1:** Comparison of different reported sensors for Pb(ii) ions detection

Nano Probe	Methods	Metal	LOD	Ref.
NaYF_4_ UCNPs and AuNPs	Fluorescence	Pb(ii)	0.020 μM	45
NaYF_4_/Yb^3+/^Tm^3+^ and CdTe QDs	Fluorescence	Pb(ii)	0.080 μM	46
ZnS QDs	Fluorescence	Pb(ii)	0.93 μM	47
NH_2_-CQDs/AuNCs	Fluorescence	Pb(ii)	0.5 μM	48
[Tb(L)(H_2_O)_5_]n MOFs	Fluorescence	Pb(ii)	0.1 μM	49
([Ln_2_(FDC)_3_DMA(H_2_O)_3_] DMA_4_·5H_2_OMOFs	Fluorescence	Pb(ii)	8.22 μM	50
Mn@ZnSe QDs	Fluorescence	Pb(ii)	29.8 μM	51
Bodipy-Si	Fluorescence	Pb(ii)	0.155 μM	This study

Tap water, drinking water and the river water supplied by Seydişehir/Konya were performed for the recovery tests in the real samples (Table 2). The spiked amounts of Pb(ii) ions in known concentration were recovered in a range of 94.6–98.7%. As result, the fluorescent adsorbent, Bodipy-Si, was effectively utilized for the detection/removal of lead(ii) ions in diverse real samples.

**Table 2:** The analysis of Pb(ii) in real water samples

Sample	Spiked Pb(ii)	Found Pb(ii)	Recovery (%)
River water	—	0	—
	15	14.2	94.6 ± 0.2
	30	29.1	97.0 ± 0.2
Tap water	—	0	—
	15	14.8	98.7 ± 0.2
	30	29.3	97.7 ± 0.3
Drink water	—	0	—
	15	14.4	96.0 ± 0.2
	30	29.0	96.7 ± 0.3

## 4. Conclusion

Consequently, we designed and prepared a fluorescent hybrid material, Bodipy-Si, for the removal and detection of Pb(ii) ions that can sensitively and selectively determine Pb(ii). So, Bodipy-Si has a low LOD value of 0.155 μM and displayed a good adsorption capacity because the triazole groups of Bodipy-Si enable an electronegative cage and easily interact with Pb(ii) ions. As all results, the fluorescent adsorbent enables both the detection and removal and the of Pb(ii) ions in wastewater. Moreover, the fluorescent hybrid surface designed as an electronic cage allows the detection and removal of toxic analytes that can be accepted as a challenge for the remediation of wastewater.

## Conflicts of interest

The authors declare no competing interests.

## Supplementary Material

RA-013-D2RA07651A-s001
