# Supplementary material for: A high-performance fluorescent hybrid material for fluorometric detection and removal of toxic Pb(ii) ions from aqueous media: performance and challenges
Source: RSC Adv. 2023 Jan 20;13(4):2683–91. doi: 10.1039/d2ra07651a (PMC9854339; doi:10.1039/d2ra07651a)
Supplement: RA-013-D2RA07651A-s001 [file RA-013-D2RA07651A-s001.pdf]

Electronic Supplementary Material (ESI) for RSC Advances.
This journal is © The Royal Society of Chemistry 2023

A high-performance fluorescent hybrid material for fluorometric detection and removal of toxic Pb (II) ion from aqueous media: performance and challenge

Abdulrazzak Abdullah, Ahmed Nuri Kursunlu[1], Ersin Güler

Department of Chemistry, Selcuk University, Campus, 42031, Konya, Türkiye

Experimental section

Chemicals and apparatus

The NMR spectra (^1H and ^{13}C) of the synthesized organic compounds were performed using a nuclear magnetic resonance spectrometer (Varian 400 MHz). The emission spectra and the linked spectroscopic measurements were performed by a Perkin Elmer LS 55 spectrofluorometer. The SEM images, FT-IR spectra and elemental analysis were performed with a HITACHI (SU5000), a Bruker Fourier Transform Infrared (ATR) and a Leco CHNS 932, respectively. The remaining lead ions in the suspension were determined by Analytic Jena, Contr AA 300 spectrophotometer using the atomic absorption values.

For the ordering of the used chemicals, various companies were preferred. Silica gel (70-230 mesh) used as both purification (column chromatography) and adsorbent materials was purchased from Fluka (Switzerland). Sodium azide, d-chloroform ($CDCl_3$), 3-aminopropyltrimethoxysilane (APTMS, 97%), propargylamine, triethylamine, 2,4-dimethyl-3-ethylpyrrole, borontrifluoride diethyl etherate, N,N-diisopropylethylamine (DIPEA), 4-(chloromethyl)benzyl chloride, sodium ascorbate, copper(II)sulphate were provided from Sigma-Aldrich. Solvents (dichloromethane, ethyl alcohol, toluene, petroleum ether (40-60%), N,N-

*Corresponding author. E-mail: ankursunlu@hotmail.com (A. N. Kursunlu)

Address: Department of Chemistry, *Science Faculty, Selcuk University, 42035, Konya, Turkey*
Tel: *+90 332 223 39 02.*

dimethylformamide) and the metal nitrate salts were purchased from Merck Company (Germany, Darmstadt).

The synthesis of compounds

The compounds were prepared a known procedure [41].

The synthesis of 8-{4-(chloromethyl)phenyl}-2,6-diethyl-4,4-difluoro-1,3,5,7-tetramethyl-4-bora-3a,4a-diaza-s-indacene

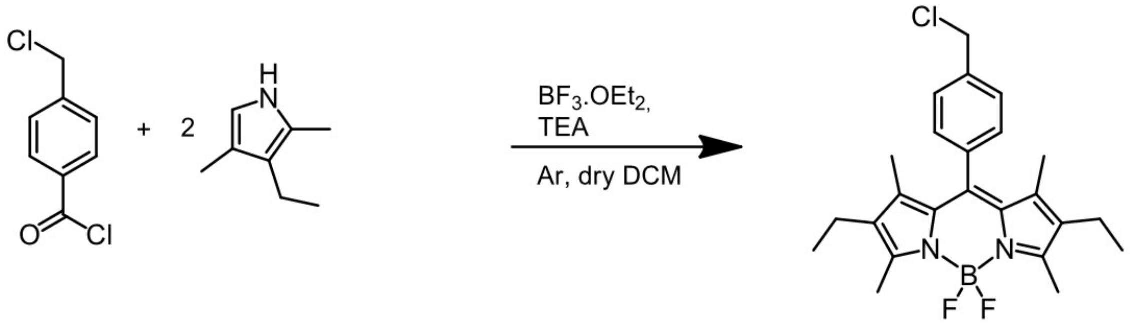

8-{4-(chloromethyl)phenyl}-2,6-diethyl-4,4-difluoro-1,3,5,7-tetramethyl-4-bora-3a,4a-diaza-s-indacene was prepared following the synthesis procedure in our previous papers. p-(chloromethyl)benzoyl chloride (7.5 g, 0.04 mol) was added to a solution (in DCM, 200 mL) of Kryptopyrrole (10.8 mL, 0.08 mol) in Ar atmosphere at room temperature. Then, the stirring of the solution was allowed for 4 h. After cooling of the solution, 10 mL of triethylamine was added to the mixture, it was stirred at r. t. for half hour and boron trifluoride diethyl etherate (8 mL) was injected by syringe. The mixture was heated to 65 ^oC for 3 hours and the residue was purified by a solution of ethylacetate-cyclohexane in 1:8 ratio (3.47 g, Yield 40%). Mp: 189 ^oC. ^1H NMR [400 MHz, $CDCl_3$]: 7.42 (PhH, *J*=8.2 Hz, d, 2H), 7.20 (PhH, *J*=8.2 Hz, d, 2H), 4.65 (CH_2, s, 2H), 2.46 (CH_3, s, 6H) 2.23 (CH_2, *J*=7.5 Hz, q, 4H) 1.29 (CH_3, s, 6H) 0.91 (CH_3, *J*=7.5 Hz, t, 6H). ^{13}C NMR [100

MHz, $CDCl_3$]: δ (ppm); 154.03, 139.49, 138.66, 135.88, 136.11, 133.02, 130.77, 128.99, 128.38, 45.66, 16.98, 14.66, 12.54, 11.66. Analytical Cal. for (%) $C_{24}H_{28}N_2F_2ClB$: H, 6.58; C, 67.22; N, 6.53; Found: H, 6.97; C, 66.98; N, 6.13. MS for $C_{24}H_{28}N_2F_2ClB$: 428 $[M+H]^+$.

The synthesis of 8-{4-(azidomethyl)phenyl}-2,6-diethyl-4,4-difluoro-1,3,5,7-tetramethyl-4-bora-3a,4a-diaza-s-indacene (Bodipy)

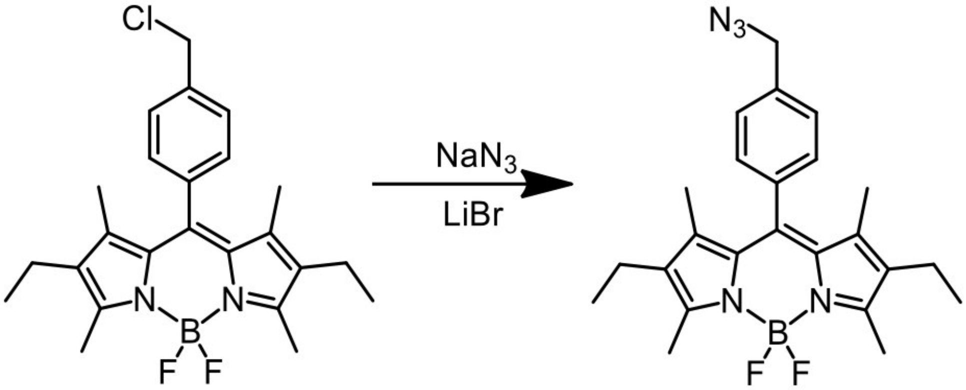

Compound **1** (0.44 mmol) and NaN_3 (0.344 g, 0.53 mol) were dissolved in N,N-dimethylformamide (20 mL) and mixed for 24 hours at room temperature. Under inert atmosphere. The residue was extracted with water/chloroform. The organic phases were collected, dried with Na_2SO_4. Following evaporation of chloroform, raw product was purified in column (only dichloromethane) (0.56 g, 96%). Caution: 3 must be handled with special care due to its explosive character, mp : 140 °C ^{1}H-NMR [400 MHz, $CDCl_3$]: 7.45 (PhH, *J*=8.0 Hz, d, 2H), 7.33 (PhH, *J*=8.1 Hz, d, 2H,) 4.48 (CH_2, s, 2H,), 2.57 (CH_3, s, 6H) 2.32 (CH_2, *J*=7.5 Hz, q, 4H), 1.28 (CH_3, s, 6H,), 0.99 (CH_3, *J*=7.5 Hz, t, 6H). ^{13}C NMR [100 MHz, $CDCl_3$]: δ (ppm); 154.04, 138.99, 138.03, 135.98, 135.62, 132.95, 130.81, 128.29, 129.03, 55.34, 17.44, 15.02, 12.32, 11.99. Analytical Cal. for (%) $C_{24}H_{28}BF_2N_5$: H, 6.48; C, 66.22; N, 16.09; Found: H, 6.67; C, 66.38; N, 16.15. MS for $C_{24}H_{28}N_5F_2B$: 435.3 $[M+H]^+$.

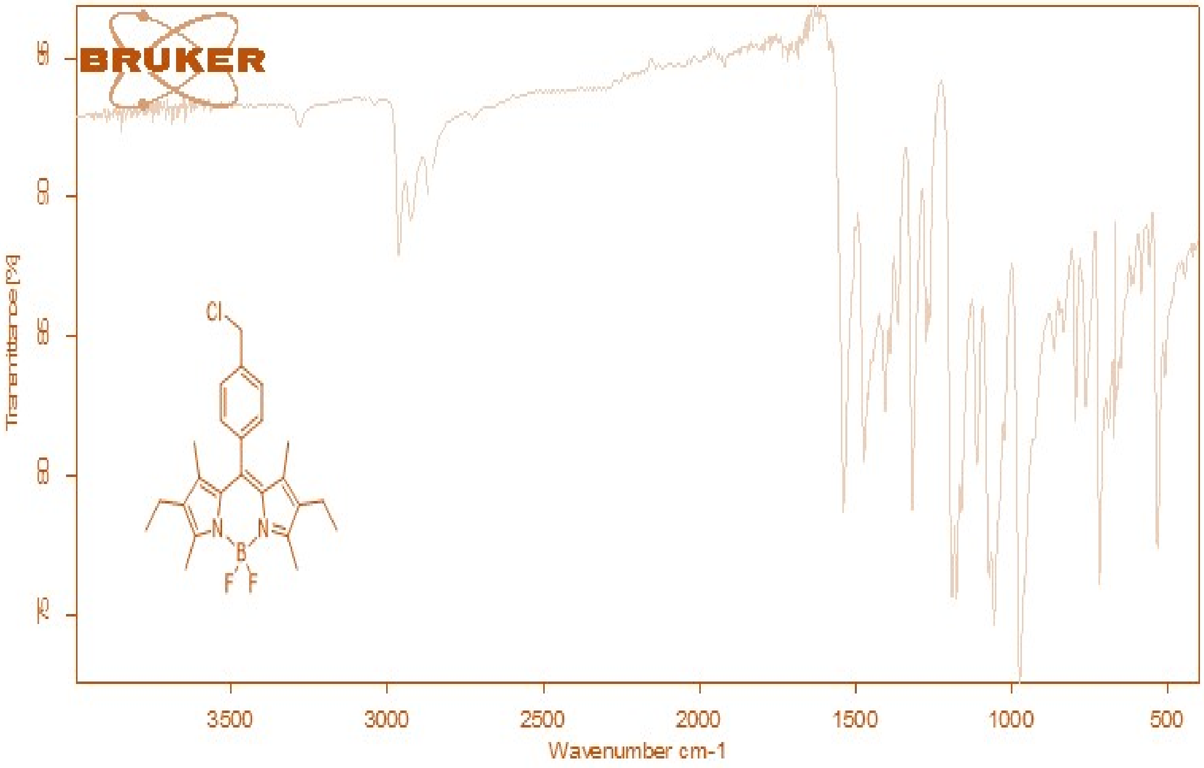

Fig.S1 FT-IR spectrum of 8-{4-(chloromethyl)phenyl}-2,6-diethyl-4,4-difluoro-1,3,5,7-tetramethyl-4-bora-3a,4a-diaza-s-indacene

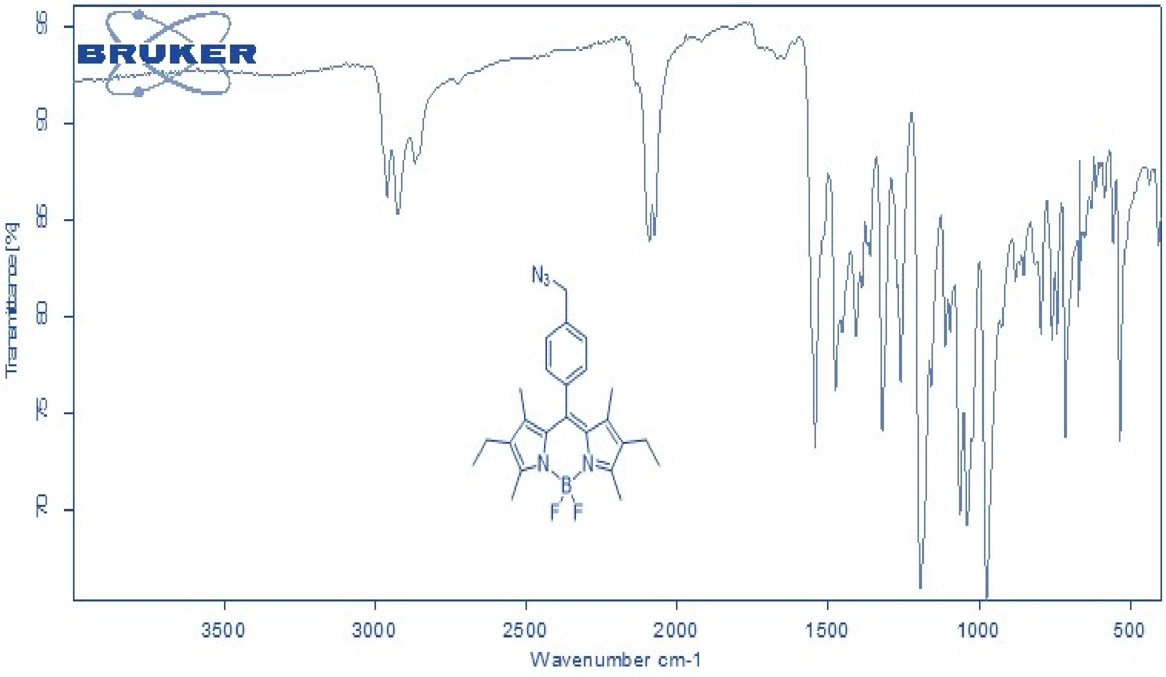

Fig. S2 FT-IR spectrum of Bodipy

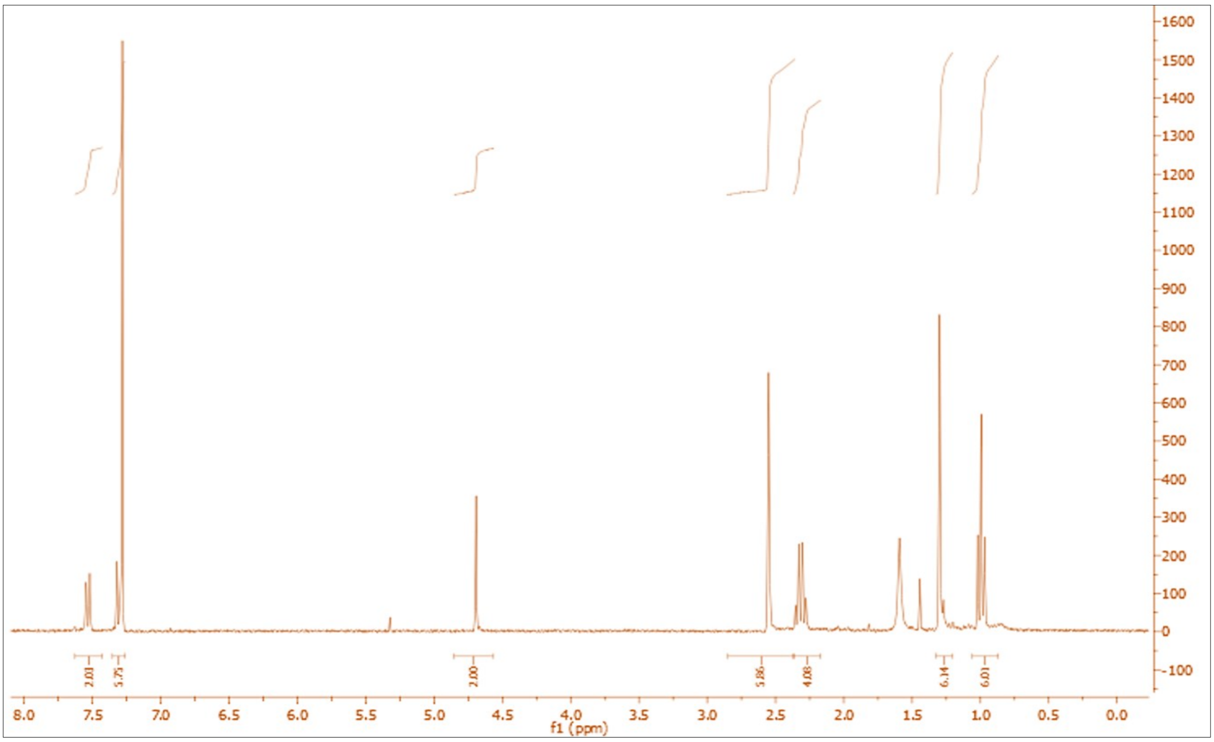

Fig.S3 1H-NMR spectrum of 8-{4-(chloromethyl)phenyl}-2,6-diethyl-4,4-difluoro-1,3,5,7-tetramethyl-4-bora-3a,4a-diaza-s-indacene

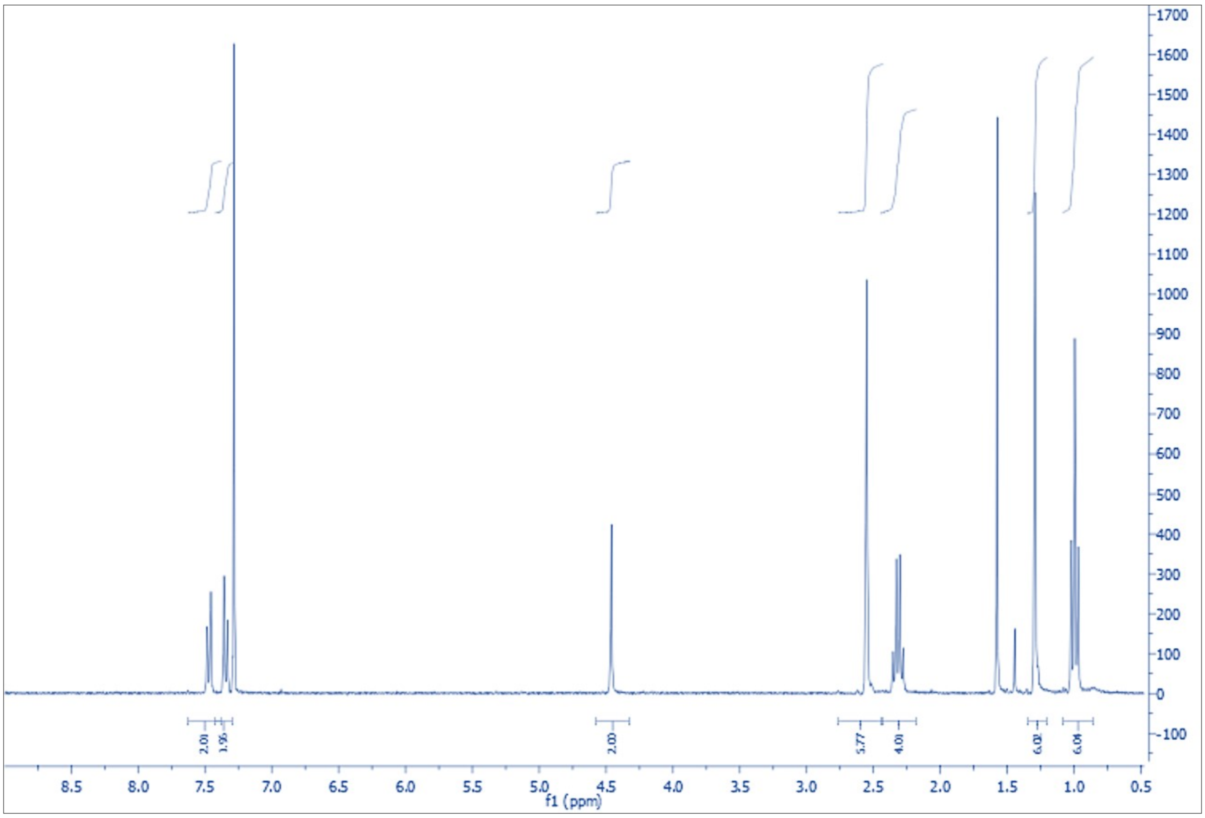

Fig.S4 ^{1}H-NMR spectrum of Bodipy (25 ^{o}C)

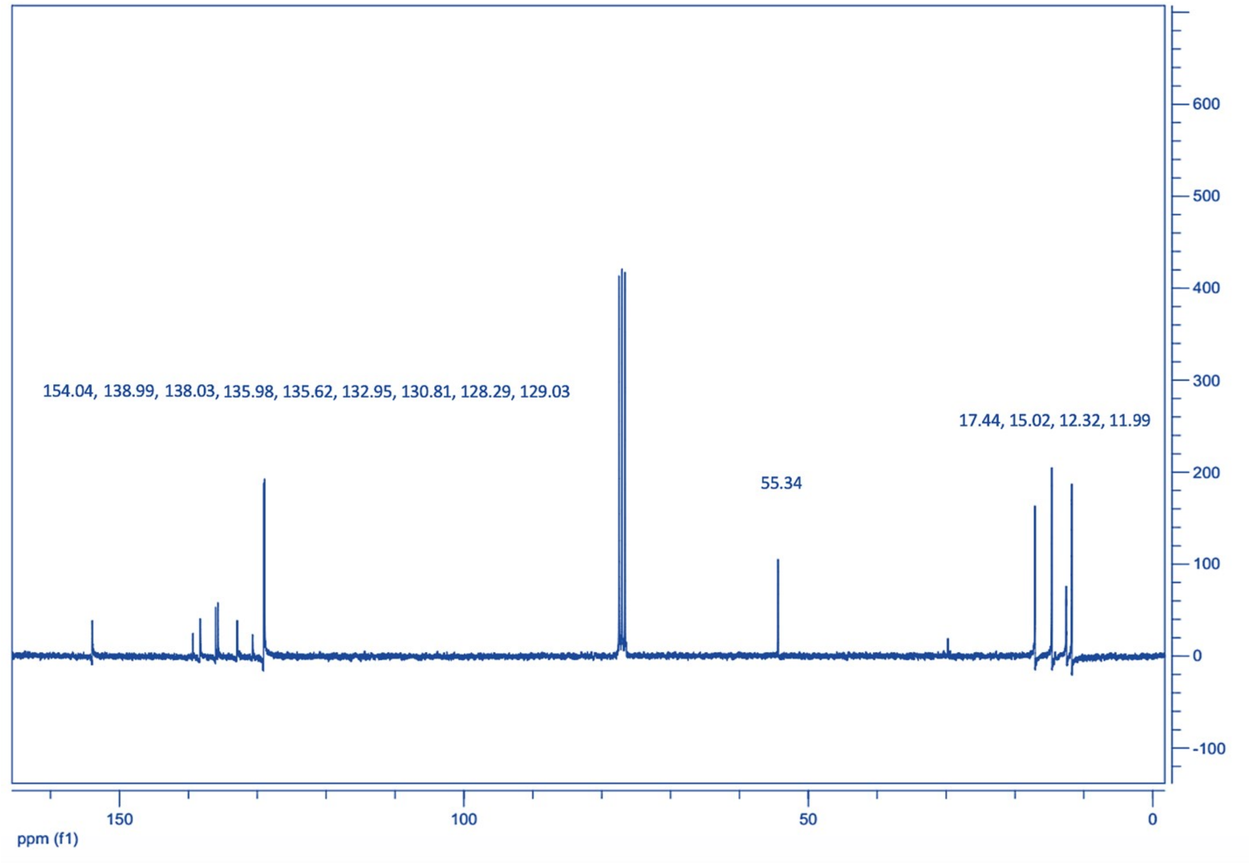

Fig.S5 ^{13}C-NMR spectrum of Bodipy (25 ^{o}C)

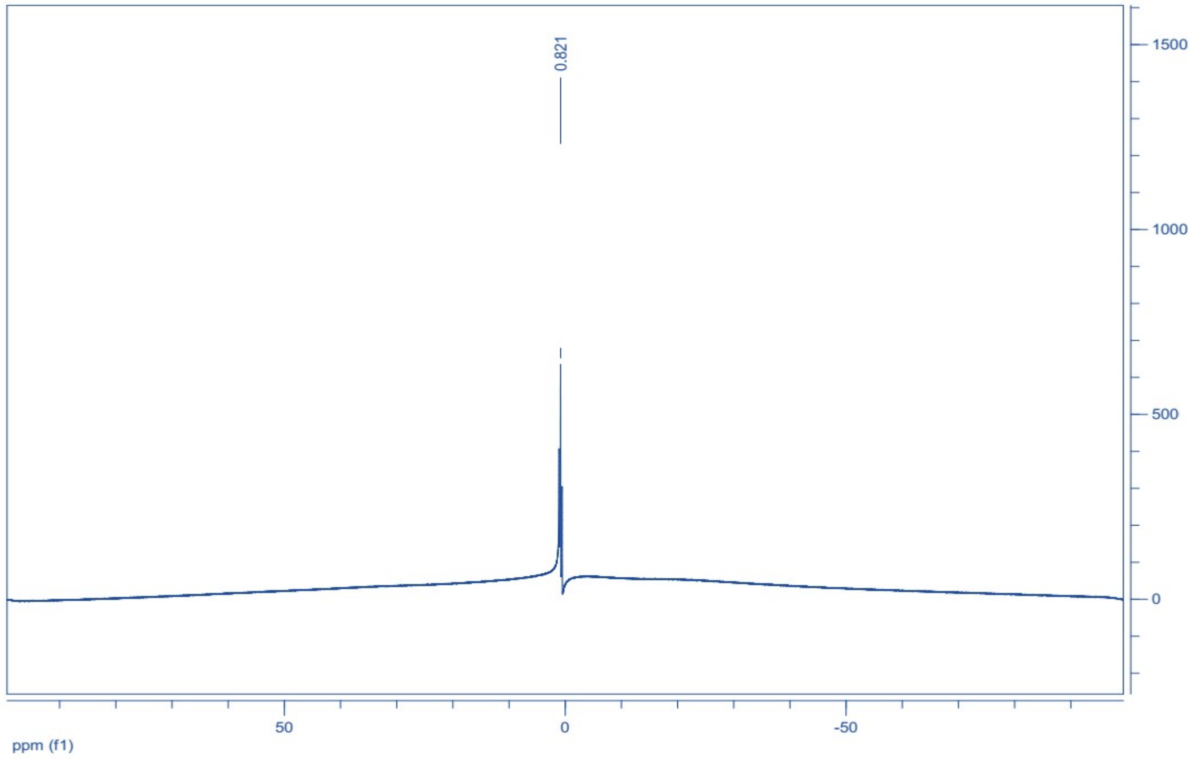

Fig

.S6 ^{11}B-NMR spectrum of Bodipy (25 ^{o}C)

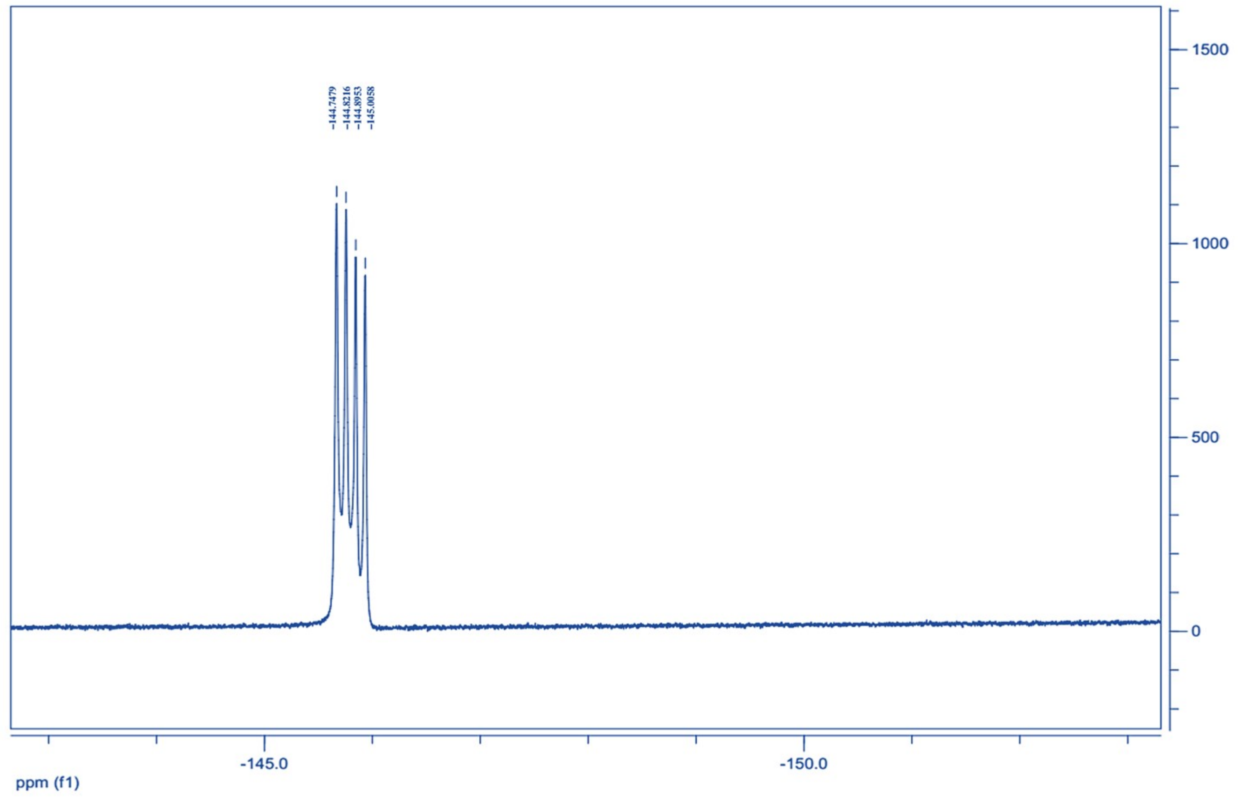

Fig.S7 ^{19}F-NMR spectrum of Bodipy (25 ^{o}C)

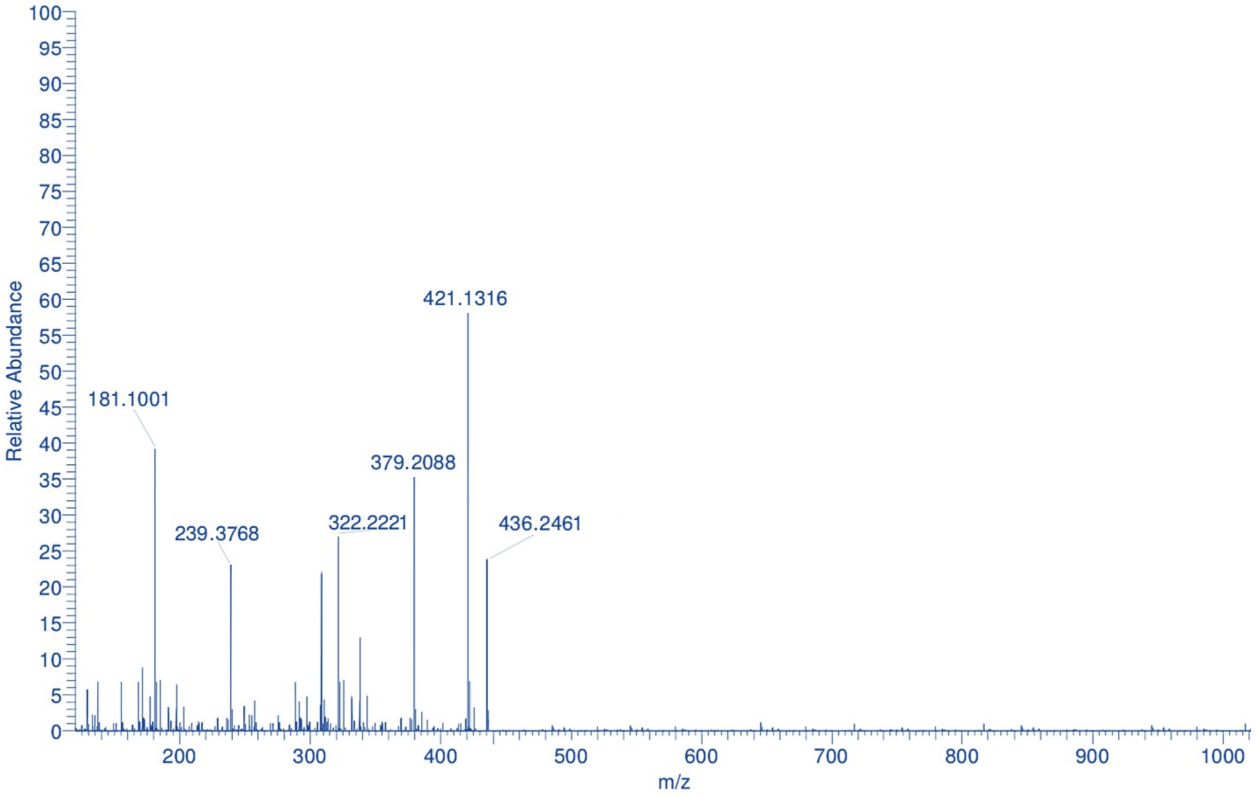

Fig.S8 Mass spectrum of Bodipy

[41] Bilgic A., Cimen A., Kursunlu A.N., 2021. Fluorescent and Easy-Make Hybrid Sensor Based-on Silica Gel & BODIPY for the Detection of Cu (II) in Aqueous Medium: Fully Characterized, Effective and Visual Data, IEEE Sensors J., 22 (3), 1882-1889.
